# Kinetics of chemical deteriorations over the frying protected by gallic acid and methyl gallate

**DOI:** 10.1038/s41598-023-38385-2

**Published:** 2023-07-08

**Authors:** Maedeh Hosseinkhani, Reza Farhoosh

**Affiliations:** Department of Food Science and Technology, Faculty of Agriculture, Ferdowsi University of Mashhad, P.O. Box 91775-1163, Mashhad, Iran

**Keywords:** Plant sciences, Chemistry

## Abstract

The present work shows the possibility of use of gallic acid (GA) and methyl gallate (MG) as natural antioxidants replacing the powerful synthetic antioxidant TBHQ in frying process. Oxidative stability index (OSI) and the kinetics of change in lipid-peroxidation conjugated dienes (LCD), carbonyls (LCO), and acid value were adopted for the evaluation purposes. GA alone (1.2 mM) and in combination with MG (75:25) provided OSI values comparable to that of TBHQ (18.5–19.0 h). The GA/MG 75:25 exerted a frying performance quite better than TBHQ (*r*_n_ = 0.1351 vs. 0.1784 h^−1^) in preventing the LCD formation. From the LCO formation standpoint, the GA/MG 75:25 (*r*_n_ = 0.0758 h^−1^) and then MG (*r*_n_ = 0.1004 h^−1^) provided better performances than TBHQ (*r*_n_ = 0.1216 h^−1^). Lipid hydrolysis was also inhibited well by GA (AV_m_ = 8.6) and GA/MG 75:25 (AV_m_ = 7.9), respectively (AV_m_ = 9.2 for TBHQ).

## Introduction

**Figure 1:**
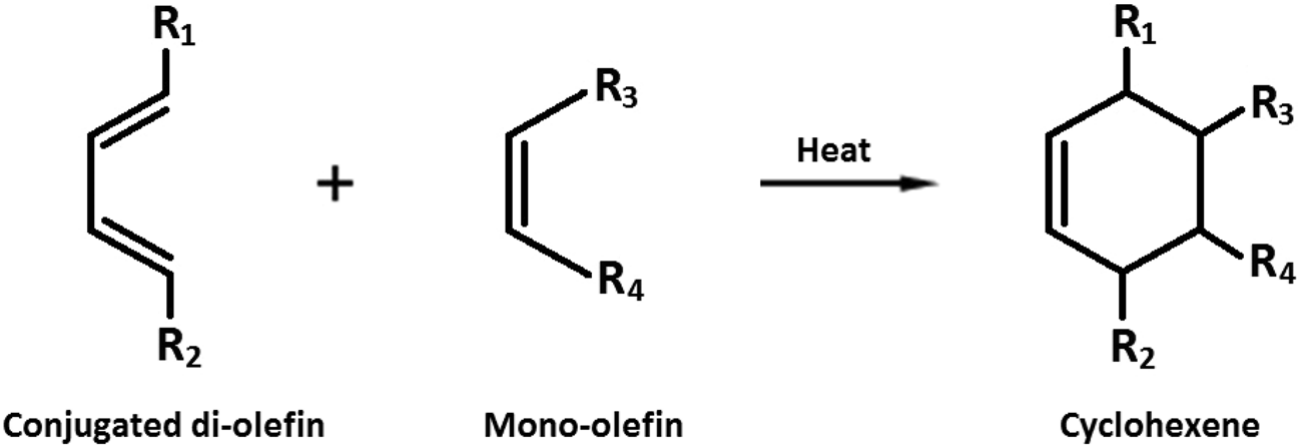
The Diels–Alder reaction.

Frying of foods in an edible oil exposed to high temperatures (170–200 °C) for a long while has always been accompanied by serious sensory and nutritional concerns related to the probable oxidative and hydrolytic deteriorations. The total content of lipid-peroxidation polar compounds is the most well-known analytical measure to evaluate the health of used frying oils^1^. However, this measure has been shown to correlate well with a simpler, quicker, and less expensive measure to determine, namely the total content of lipid-peroxidation conjugated dienes (LCD)^2^. LCD are basically a range of the primary oxidation products resulting from the double bond shifts in polyunsaturated fatty acids^3^. Their content at the initial stage of the reaction is roughly the same as that of the total conjugated and non-conjugated hydroperoxides. After prolonged frying times, however, the total LCD content becomes smaller than the total content of the conjugated and non-conjugated isomers, due to the secondary LCD oxidation leading to the loss of conjugation^4^. Moreover, the total LCD content reaches a plateau resulting from the Diels–Alder reaction (Fig. 1), which is a dimerization between a conjugated di-olefin and a mono-olefin group to form a cyclohexene ring^5^. Such a pattern is in line with the kinetic model of the accumulation of lipid hydroperoxides developed recently by the author (see the section “Kinetic data analyses”)^6,7^.

From a sensory as well as a nutritional point of view, the total content of lipid-peroxidation carbonyls (LCO), comprising a numerous variety of volatile and non-volatile secondary oxidation products, has been considered as a valuable quantitative measure to evaluate the quality of used frying oils^8^. A sigmoidal pattern has frequently been observed for the change in the total LCO content during frying, namely an initial slow increasing phase followed by a rapid rising phase terminated to a maximum value. Afterwards, its level show constant or reduced amounts^8,9^ due basically to the further degradations of the primary carbonyls to non-carbonyls and/or more volatile products^8–10^.

Acid value (AV) is the other well-known frying measure to detect the progressive hydrolysis of triacylglycerols to free fatty acids and glycerol^11^. Lower levels of AV make frying oils less prone to the strong off-flavors caused by the degradation volatile and/or non-volatile products arising from the free fatty acids, which are inherently of higher oxidative reactivity than the parent triacylglycerols^12^. Furthermore, the quite toxic component acrolein (an oral LD_50_ in rats of only 46 mg/kg of body weight) with a very pungent, irritating smell^13^ is produced from the dehydration of glycerol easily at temperatures as low as 180 °C^14^ and more quickly at higher temperatures reaching the smoke point at which an oil starts to smoke^13^. Acrolein is so volatile (boiling point 52 °C) and does not significantly remain in frying oil^13^ but its trace amounts clearly appear as the blue haze above the smoking oil^14^.

Incorporating antioxidants into the matrix of frying oils has frequently been adopted as a major treatment to protect them from the deteriorative reactions of oxidative and/or hydrolytic natures. Synthetic antioxidants, including butylated hydroxyanisole (BHA), butylated hydroxytoluene (BHT), propyl gallate (PG), and *tert*-butylhydroquinone (TBHQ), have often been used to enhance the frying performance of edible oils^15^. TBHQ has also been recognized to be the most powerful one employed widely in the food industry as well as highly resistant to thermal decomposition and/or volatilization^16^. Nevertheless, synthetic antioxidants have been questioned due to their probable contribution to increasing health risks such as cancer and carcinogenesis^17^. Addition of natural antioxidants, hence, has always been considered to be a safer and consumer-friendly way to improve the frying performance of edible oils.

The two natural phenolic compounds gallic acid (3,4,5-trihydroxybenzoic acid, GA) and methyl gallate (MG) have fully been recognized due to many of their valuable biological effects^18,19^. A limited number of studies in recent years have indicated the powerful antioxidant activity of GA and MG in some storage and rather harsh conditions^20–23^. However, there is no analytical data on their protective effect under frying processes. Hence, this study aimed to investigate the kinetics of oxidative and hydrolytic deteriorations over a frying process protected by GA and MG compared with the powerful synthetic antioxidant TBHQ.

## Materials and methods

### Materials

Refined, bleached, and deodorized sunflower and palm olein oils with no added antioxidants were supplied by Segol factory in Nishabour, Iran. The oil samples were stored at − 18 °C until analysis. Agria potato variety was purchased from the farmers in Fariman, Iran. All the chemicals and solvents used in the study were of analytical reagent grade and purchased from Merck (Darmstadt, Germany) and Sigma-Aldrich (St. Louis, MO, USA).

### Deep-fat frying procedure

Peeled and cut (4.0 × 0.5 × 0.5 cm) potatoes were submerged in water (25 °C) until frying. After rinsing with cold water and drying by a fan as well as a clean towel, they (30 g) were fried (180 °C) in 1 L of the oil treatments (sunflower/palm olein 65:35, as control oil, containing a total concentration of 1.2 mM of TBHQ, GA, MG, or GA/MG in the ratios of 75:25, 50:50, and 25:75) with no replenishment by a bench-top fryer (Hamilton DF-535T, PRC). The potato pieces were fried at 5-min intervals for 8 h. At 1 h intervals, 45 g of the oils were filtered into a screw-cap vial and immediately stored in the dark at − 3 °C until analysis. Frying processes were carried out in four replications^8^.

### Chemical properties of the frying oil

To determine fatty acid composition, fatty acid methyl esters (FAME) were prepared by shaking a solution of the oil sample in hexane (0.3 g in 7 mL) with 7 mL of 2 N methanolic KOH at 50–55 °C for 15 min. After settling the solution for 5 min, the upper layer was mixed with anhydrous Na_2_SO_4_ and then filtered. FAME were injected into a gas–liquid chromatograph (Hewlett-Packard, Santa Clarita CA, USA) equipped with a FID and a BPX 70 capillary column (60 m × 0.22 mm I.D., 0.2 mm film thickness), using He as the carrier gas at a flow rate of 0.7 mL min^−1^. The oven temperature was maintained at 198 °C and those of the injector and detector at 280 °C and 250 °C, respectively. The analysis was carried out in duplicate and data was reported as relative area percentages^24^.

Peroxide value (PV), expressed as milliequivalents of O_2_ per kilogram of the oil sample, was measured according to the thiocyanate method described by Shantha and Decker^25^. Total tocopherols (TT) content was measured according to the colorimetric method described by Wong et al.^26^. A calibration curve of α-tocopherol in toluene was prepared in a concentration range of 0–240 μg mL^−1^. TT content was reported as milligrams of α-tocopherol per kilogram of the oil sample. Total phenolics (TP) content was measured spectrophotometrically using Folin–Ciocalteau’s reagent as described by Capannesi et al.^27^. A calibration curve of GA in CH_3_OH was prepared in a concentration range of 0.04–0.40 mg mL^−1^. TP content was reported as milligrams of GA per kilogram of the oil sample.

### Oxidative stability index (OSI)

A Metrohm Rancimat model 743 (Herisau, Switzerland) was used to measure OSI (h). The tests were carried out with 3 g of the oil samples at 110 °C and an airflow rate^28^ of 15 L h^−1^.

### Total LCD content

The oil samples were dissolved in hexane (1:600) and their absorbance were read at 234 nm against HPLC grade hexane as blank. An extinction coefficient of 29,000 mol/L was used to calculate millimoles of LCD per liter^29^.

### Total LCO content

The content of total LCO was measured according to the method developed by Endo et al.^30^ using 2-propanol and 2,4-decadienal as solvent and standard, respectively. Total LCO content was reported as micromoles of 2,4-decadienal per gram of the oil sample.

### Acid value (AV)

Ten grams of the oil samples were dissolved in 50 ml of chloroform-ethanol (50:50 v/v) and titrated with 0.1 N ethanolic KOH. AV was the milligrams of KOH required to neutralize the free fatty acids in 1 g of the oil sample^31^.

### Kinetic data analyses

Kinetic curves of LCD accumulation were drawn by plotting the changes in the total content of LCD (mM) versus time *t* (h). The sigmoidal Eq. (1) was fitted on the kinetic data points of LCD accumulation^6,7^:

1 $${\text{LCD}} = \frac{a}{{b + e^{a(c - t)} }}$$

where *a*, *b*, and *c* are the equation parameters. The finite value LCD_max_ is calculated from the ratio *a*/*b*. The maximum rate of LCD accumulation (*r*_max_, mM h^−1^) equals 0.25*a*^2^/*b*. Its normalized form (*r*_max_/LCD_max_ = *r*_n_, h^−1^) is given by 0.25*a*. The parameter *b* represents the pseudo-second order rate constant *k*_d_ (mM^−1^ h^−1^) for the LCD lost through chemical decomposition or the Diels–Alder reaction. The time at which the total content of LCD practically approaches LCD_max_ (*t*_max_, h) is obtained from the ratio (2 + *ac − *ln*b*)/*a*.

The method developed recently by the author was employed to calculate the LCO-based kinetic parameters^32^. Kinetic curves of LCO (μmol g^−1^) accumulation were drawn by plotting the changes in the total content of carbonyls versus time *t* (h). The sigmoidal Eq. (2) was fitted on the kinetic data points of LCO accumulation:

2 $${\text{LCO}} = a + \frac{b}{{1 + e^{{\left( {\frac{c - t}{d}} \right)}} }}$$

where *a*, *b*, *c*, and *d* are the equation parameters. The finite value LCO_max_, where the rate of LCO accumulation reaches zero at infinity, equals *a* + *b*. At the equation’s turning point with the coordinates *t*_T_ = *c* and LCO_T_ = *a* + 0.5*b*, the rate of LCO accumulation (μmol g^−1^ h^−1^) reaches the maximum value *r*_max_ = 0.25*b*/*d*. Its normalized form (*r*_max_/LCO_max_ = *r*_n_, h^−1^) is calculated from the ratio *b*/4*d*(*a* + *b*). The time at which carbonyls practically approach LCO_max_ (*t*_max_, h) is obtained from *c* + 2*d*.

Kinetic curves of organic acids (mg g^−1^) accumulation were drawn by plotting the changes in AV versus time *t* (h). The power Eq. (3) was fitted on the kinetic data points:

3 $${\text{AV}} = a + bt^{c}$$

where *a*, *b*, and *c* are the equation parameters. From a mathematical point of view, the ratio 1/*bc* (AV_m_), which quantitatively represents the change pattern of organic acids over time, was used as an empirical measure of hydrolytic stability of the frying system.

### Statistical analysis

All determinations were carried out in triplicate and data were subjected to analysis of variance (ANOVA). ANOVA and regression analyses were performed according to the MStatC (developed by the Department of Plant Science and Soil of Michigan State University, USA) and SlideWrite version 7.0 (Advanced Graphics Software, Inc., Toronto, Canada), respectively. Significant differences between means were determined by Duncan’s multiple range tests. P values less than 0.05 were considered statistically significant.

## Results and discussion

### Chemical composition of the oil samples

**Figure 2:**
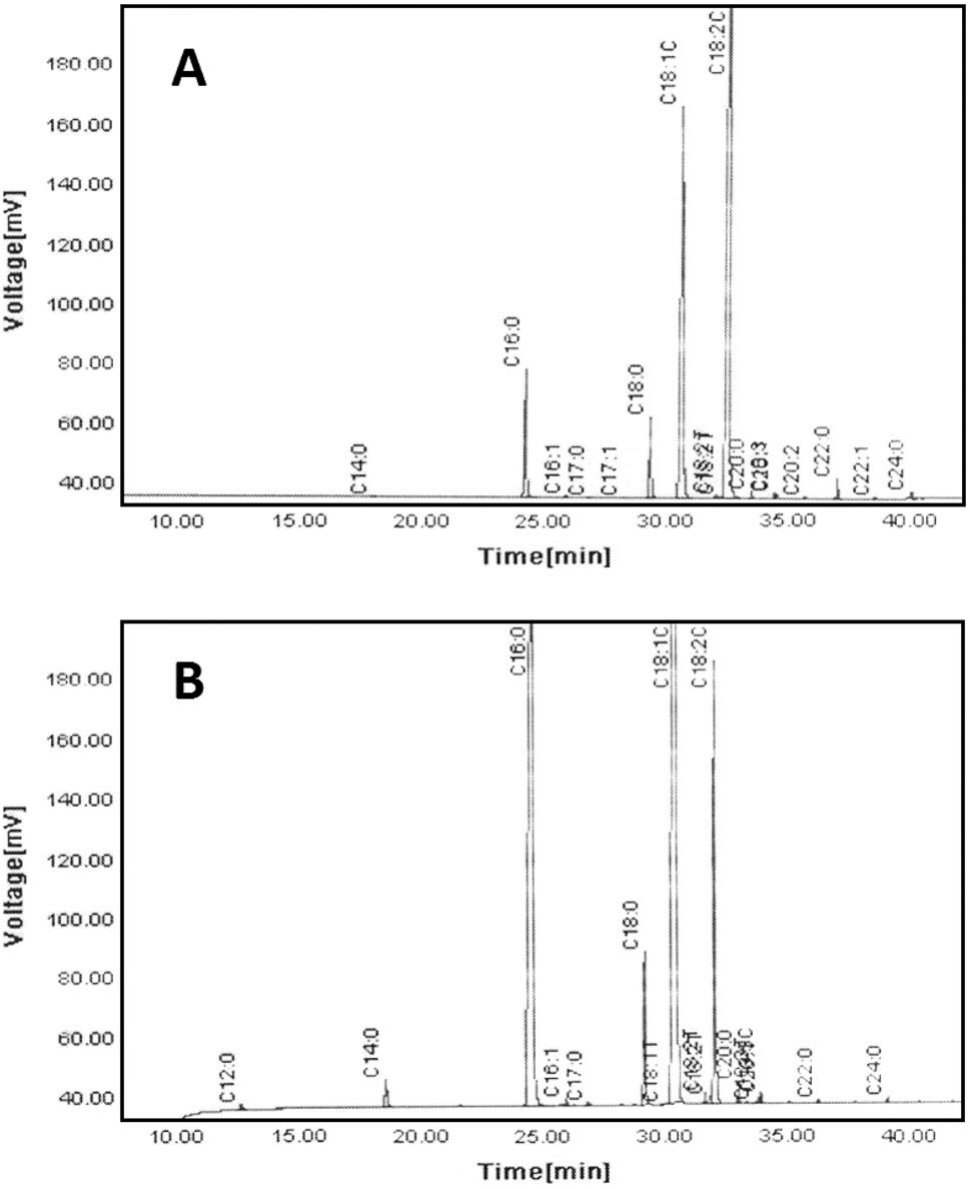
Gas–liquid chromatograms of the sunflower (**A**) and palm olein (**B**) oils.

Table 1 shows the fatty acid composition (Fig. 2), the initial quality indicators (PV and AV), and the TT and TP contents of the oils studied. The oil samples had the fatty acid compositions in agreement with those usually reported in literature. They could be differentiated from each other by mainly the contents of palmitic (C16:0), oleic (C18:1), and linoleic (C18:2) acids. Due to the considerably higher levels of C16:0 (38.9 vs. 6.95%) and C18:1 (42.0 vs. 25.5%), the palm olein oil was of about 3.7- and 1.6-fold contents of saturated (SFA) and monounsaturated (MUFA) fatty acids, respectively, compared with the sunflower oil. In contrast, the sunflower oil possessed a polyunsaturated fatty acids (PUFA, mainly C18:2: 61.8 vs. 12.2%) content of almost five times that of the palm olein oil. This naturally makes the sunflower oil a rather unstable frying medium with respect to the relative rate of oxidation for C18:3, C18:2, C18:1, and C18:0 as 2500:1200:100:1^33^. Therefore, a blend of the sunflower and palm olein oils (65:35), indicating better qualitative parameters than each of the oils alone over frying in overall^34^, was used to evaluate the frying performance of the antioxidant treatments.

**Table 1:** Chemical properties of the sunflower oil, palm olein, and their blend.

Chemical property	Oil sample		
	Sunflower	Palm olein	Sunflower/Palm olein 65:35
Fatty acids (%w/w)			
C12:0	–	0.22 ± 0.00	0.08 ± 0.00
C14:0	0.08 ± 0.00	1.03 ± 0.02	0.41 ± 0.01
C16:0	6.95 ± 0.32	38.9 ± 0.2	18.1 ± 0.2
C18:0	3.83 ± 0.09	4.37 ± 0.07	4.02 ± 0.06
C18:1	25.5 ± 0.3	42.0 ± 0.6	31.3 ± 0.3
C18:2	61.8 ± 0.4	12.2 ± 0.1	44.4 ± 0.3
C18:3	0.22 ± 0.01	0.27 ± 0.01	0.24 ± 0.01
C20:0	0.26 ± 0.00	0.36 ± 0.04	0.30 ± 0.01
C20:1	0.16 ± 0.00	0.16 ± 0.01	0.16 ± 0.0
C20:2	0.03 ± 0.00	–	0.02 ± 0.00
C22:0	0.76 ± 0.06	0.08 ± 0.00	0.52 ± 0.04
C22:1	0.04 ± 0.00	–	0.03 ± 0.00
C24:0	0.25 ± 0.00	–	0.16 ± 0.00
SFA	12.1 ± 0.2	45.0 ± 0.1	23.6 ± 0.14
MUFA	25.7 ± 0.3	42.2 ± 0.5	31.5 ± 0.3
PUFA	62.1 ± 0.3	12.5 ± 0.1	44.7 ± 0.2
PV (meq kg^−1^)	1.53 ± 0.01	0.40 ± 0.00	1.14 ± 0.00
AV (mg g^−1^)	0.15 ± 0.01	0.26 ± 0.03	0.19 ± 0.04
TT (mg kg^−1^)	490 ± 1	185 ± 1	383 ± 1
TP (mg kg^−1^)	36.0 ± 1.0	53.1 ± 0.2	42.0 ± 0.7

*SFA* saturated fatty acids, *MUFA* monounsaturated fatty acids, *PUFA* polyunsaturated fatty acids, *PV* peroxide value, *AV* acid value, *TT* total tocopherols content, *TP* total phenolics content.

The PV and AV of the oils and their blend were all less than 2 meq kg^−1^ and 0.3 mg g^−1^, respectively, reflecting that they were unoxidized and of high initial quality (Table 1). The indigenous tocopherols and phenolic compounds, which are basically recognized for their biologically useful effects as well as antioxidant activities, were within their normal quantitative range in the corresponding refined, bleached, and deodorized oils^35^.

### OSI values

**Figure 3:**
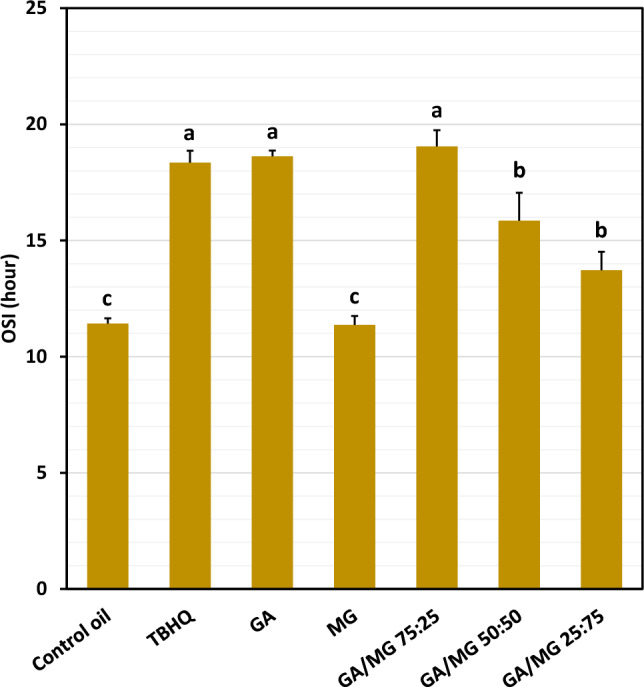
Oxidative stability index (OSI, h) of the control oil (sunflower/palm olein 65:35) in the presence of 1.2 mM of *tert*-butylhydroquinone (TBHQ) or gallic acid (GA)/methyl gallate (MG) combinations. Means ± SD (standard deviation) with the same lowercase letters are not significantly different at *p* < 0.05.

The control oil showed an OSI value of 11.4 h (Fig. 3), indicating its time resistance to the dramatically increased formation of some volatile acids (mostly formic acids, with lesser amounts of acetic, propionic, and other acids) as secondary oxidation products at thermally (~ 110–130 °C) and oxidatively (bubbling air into the oil) harsh conditions^36^. Interestingly, GA provided an OSI value comparable to that of the powerful synthetic antioxidant TBHQ (~ 18.5 h). However, its methyl ester derivative MG was not able to significantly stabilize the control oil. Replacing 25 percent of the GA concentration with MG insignificantly increased the value of OSI to 19.0 h, although the higher MG percentages led to the reduced OSI values 15.9 and 13.7 h for the GA/MG combinations 50:50 and 25:75, respectively.

### Kinetics of change in the total LCD and LCO contents and AV

**Figure 4:**
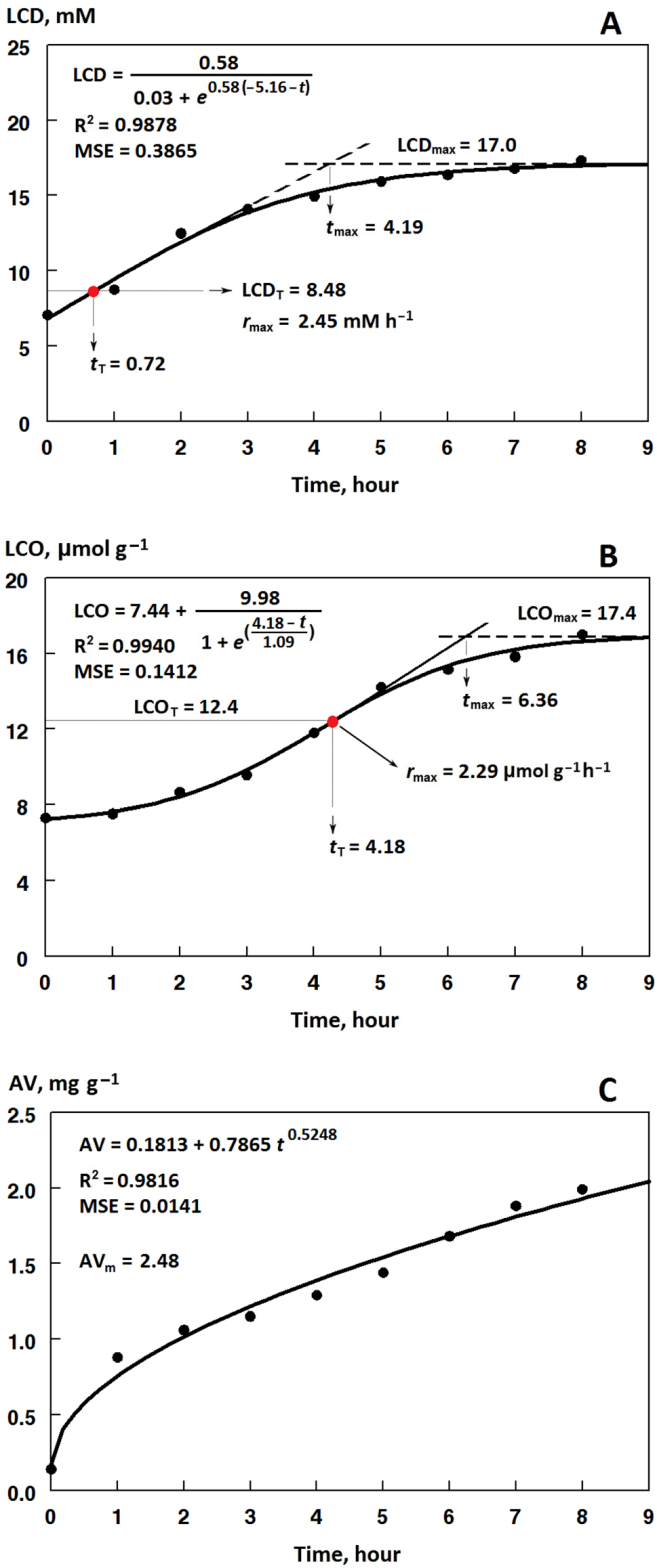
Kinetic curves of the accumulation of lipid-peroxidation conjugated dienes (LCD, **A**), carbonyls (LCO, **B**), and organic acids (AV, **C**) during the frying of the control oil (sunflower/palm olein 65:35) containing 1.2 mM of gallic acid/methyl gallate (50:50) at 180 °C, and the kinetic parameters from the Eqs. (1)–(3) fitted on the whole range of the data points. LCO_T_/LCD_T_: total LCO/LCD contents at the turning point of the sigmoidal equations with the x-coordinates *t*_T_; LCO_max_/LCD_max_: the maximum total LCO/LCD contents attained at the values of *t*_max_; *r*_max_: maximum rate of LCO/LCD accumulations; AV_m_: the quantitative measure of hydrolytic stability of the frying system.

**Figure 5:**
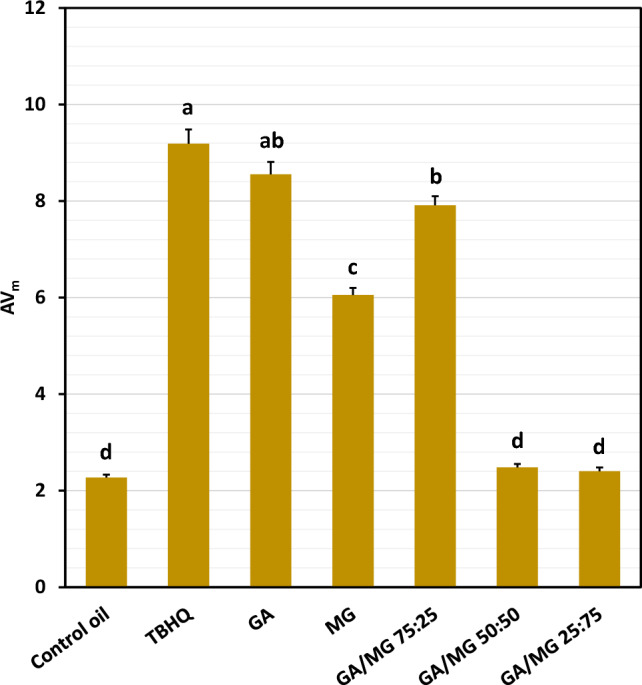
The quantitative measure of hydrolytic stability (AV_m_) resulted from the accumulation curve of lipid-peroxidation organic acids over the frying of the control oil (sunflower/palm olein 65:35) at 180 °C in the presence of 1.2 mM of *tert*-butylhydroquinone (TBHQ) or gallic acid (GA)/methyl gallate (MG) combinations. Means ± SD (standard deviation) with the same lowercase letters are not significantly different at *p* < 0.05.

The sigmoidal (1 and 2) and power (3) equations fitted very well (R^2^ > 0.97) the changes in the total LCD and LCO contents and AV, respectively, over the frying process at 180 °C (Fig. 4A–C). The kinetic data resulted from the corresponding accumulation curves are shown in Tables 2, 3, and Fig. 5, respectively.

**Table 2:** The kinetic data resulted from the accumulation curve of the total content of lipid-peroxidation conjugated dienes (LCD, mmol L^−1^) over the frying time (*t*, h) of the control oil (sunflower/palm olein 65:35) at 180 °C in the presence of 1.2 mM of *tert*-butylhydroquinone (TBHQ) or gallic acid (GA)/methyl gallate (MG) combinations.

Oil treatment	Kinetic parameter				
	LCD_max_ (mM)	*t*_max_ (h)	*r*_max_ (mM h^−1^)	*k*_d_ (mM^−1^ h^−1^)	*r*_n_ (h^−1^)
Control oil	27.8 ± 1.7^a^	3.72 ± 0.05^a^	5.81 ± 0.22^a^	0.0303 ± 0.0041^b^	0.2095 ± 0.0071^a^
TBHQ	15.5 ± 0.6^b^	3.12 ± 0.04^e^	2.76 ± 0.08^b^	0.0461 ± 0.0020^a^	0.1784 ± 0.0014^b^
GA/MG					
100:0	16.5 ± 0.5^b^	4.15 ± 0.10^c^	2.45 ± 0.08^cd^	0.0362 ± 0.0010^b^	0.1488 ± 0.0014^d^
75:25	16.4 ± 0.6^b^	4.52 ± 0.03^b^	2.22 ± 0.08^d^	0.0329 ± 0.0015^b^	0.1351 ± 0.0021^e^
50:50	17.0 ± 0.4^b^	4.19 ± 0.20^b^	2.45 ± 0.09^cd^	0.0344 ± 0.0028^b^	0.1443 ± 0.0036^d^
25:75	16.5 ± 0.2^b^	3.92 ± 0.02^d^	2.49 ± 0.02^c^	0.0367 ± 0.0011^b^	0.1511 ± 0.0028^d^
0:100	16.8 ± 1.3^b^	3.75 ± 0.10^d^	2.82 ± 0.12^b^	0.0405 ± 0.0037^ab^	0.1686 ± 0.0046^c^

Means ± SD (standard deviation) within a column with the same lowercase letters are not significantly different at *p* < 0.05. LCD_max_: The maximum total LCD content attained during the frying process; *t*_max_: The time at which conjugated dienes practically approach LCD_max_; *r*_max_: Maximum rate of LCD accumulation; *k*_d_: The pseudo-second order rate constant of the LCD lost through chemical decompositions or the Diels–Alder reaction; *r*_n_: Normalized *r*_max_.

**Table 3:** The kinetic data resulted from the accumulation curve of the total content of lipid-peroxidation carbonyls (LCO, μmol g^−1^) over the frying time (*t*, h) of the control oil (sunflower/palm olein 65:35) at 180 °C in the presence of 1.2 mM of *tert*-butylhydroquinone (TBHQ) or gallic acid (GA)/methyl gallate (MG) combinations.

Oil treatment	Kinetic parameter			
	LCO_max_ (μmol g^−1^)	*t*_max_ (h)	*r*_max_ (μmol g^−1^ h^−1^)	*r*_n_ (h^−1^)
Control oil	30.8 ± 1.2^a^	7.79 ± 0.18^b^	4.04 ± 0.09^a^	0.1317 ± 0.0043^a^
TBHQ	15.3 ± 0.9^d^	5.40 ± 0.21^d^	1.86 ± 0.08^c^	0.1216 ± 0.0079^a^
GA/MG				
100:0	17.1 ± 0.9^d^	6.52 ± 0.30^c^	2.09 ± 0.15^bc^	0.1225 ± 0.0068^a^
75:25	23.3 ± 1.6^b^	10.7 ± 0.8^a^	1.76 ± 0.07^c^	0.0758 ± 0.0076^c^
50:50	17.4 ± 1.4^cd^	6.36 ± 0.29^c^	2.29 ± 0.11^b^	0.1321 ± 0.0088^a^
25:75	17.2 ± 1.7^cd^	6.27 ± 0.28^c^	2.16 ± 0.09^b^	0.1253 ± 0.0075^a^
0:100	20.7 ± 1.3^bc^	7.37 ± 0.24^b^	2.08 ± 0.07^b^	0.1004 ± 0.0057^b^

Means ± SD (standard deviation) within a column with the same lowercase letters are not significantly different at *p* < 0.05. LCO_max_: The maximum total LCO content attained during the frying process; *t*_max_: The time at which carbonyls practically approach LCO_max_; *r*_max_: Maximum rate of LCO accumulation; *r*_n_: Normalized *r*_max_.

The control oil was of the significantly highest value of LCD_max_ (Table 2), which the level of that is expected to be affected by the rates of LCD formation (represented in overall by the value of *r*_max_) and LCD loss through chemical decompositions and/or the Diels–Alder reaction (represented in overall by the value of *k*_d_). The maximum level of total conjugated and non-conjugated lipid hydroperoxides has been shown to correlate very well with the *r*_max_/*k*_d_ ratio^7^. The powerful synthetic antioxidant TBHQ significantly decreased the value of LCD_max_, being consistent with the remarkably reduced *r*_max_ value. There was no significant difference between the LCD_max_ value of TBHQ and those of all the GA/MG combinations. However, GA and its combinations with MG provided the significantly lower values of *r*_max_ compared with TBHQ. The value of *r*_n_, which unifies the two kinetic parameters, provided a better oxidative measure to compare the frying performance of the antioxidants. On this basis, as shown in Table 2, all the GA/MG combinations with a better synergistic effect observed for the GA/MG 75:25 treatment demonstrated frying performances significantly higher than that found for TBHQ. Moreover, the value of *t*_max_ as a time parameter indicating the resistance against the formation of conjugated dienes revealed the best frying performance for the GA/MG 75:25 treatment as well.

The kinetics of change in carbonyls were found to be similar, to a large extent, to those in conjugated dienes in the presence of the antioxidants. As shown in Table 3, the significantly highest LCO_max_ value of the control oil was almost halved by TBHQ, which was in line with the corresponding reduced value of *r*_max_. However, the GA/MG 75:25 treatment and then MG alone provided better performances than TBHQ against the progressive generation of carbonyls over the frying process. This can clearly be seen in their significantly lower and higher values of *r*_n_ and *t*_max_, respectively. Considering roughly the same *r*_max_ values, the better performances of the formers could be explained by their lower degradation rates of primary carbonyls to the fragments of lower molecular weight, escaping more easily from the system, or to non-carbonyls undetectable by the LCO assay^8–10^.

Figure 5 exhibits the empirical measure of hydrolytic stabilities AV_m_ for the control oil as affected by the antioxidant treatments. TBHQ and GA with no statistically significant difference were the most efficient antioxidants to prevent hydrolysis of the frying medium and, as a result, the following nutritional and toxicological degradations^12,13^. Although MG significantly improved the hydrolytic resistance of the control oil, it did not exert the same anti-hydrolysis activity as GA did. Besides, increasing its contribution to the GA/MG treatments decreased the ability of GA in protecting the frying medium against hydrolysis.

## Conclusions

The present study was the first attempt to kinetically evaluate frying performance of the two well-known natural antioxidants gallic acid (GA) and methyl gallate (MG). Given the inhibition of the primary and secondary oxidation products during the frying process, GA alone and in combination with its methyl ester (GA/MG 75:25) exerted the same or even better frying performances compared with the powerful synthetic antioxidant TBHQ. Lipid hydrolysis was also inhibited very well by the natural antioxidants. This enables edible oil industry to supply healthy frying oils of reduced risk due to replacing TBHQ with the natural ones, although some complimentary studies in a number of different frying media used to fry some other foodstuffs could be helpful for a better assessment of the efficiency of GA and MG.

## Data Availability

All data generated or analysed during this study are included in this published article.
